# Data management during COVID-19 outbreak response in Ghana: a reference laboratory perspective on key issues and measures

**DOI:** 10.4314/gmj.v55i2s.8

**Published:** 2021-06

**Authors:** Evangeline Obodai, George B Kyei, James Aboagye, Evelyn Y Bonney, Ivy A Asante, Joseph K H Bonney, Mildred Adusei-Poku, Helena Lamptey, Bright Adu, Ernest Kenu, Kwadwo A Koram, William K Ampofo, John K Odoom

**Affiliations:** 1 Virology Department. Noguchi Memorial Institute for Medical Research, College of Health Sciences, University of Ghana. Off Akilagpa Sawyerr Road. PO Box LG 581, Legon, Accra, Ghana; 2 Department of Medicine, Washington University School of Medicine in St. Louis, 660 S. Euclid Avenue, St Louis, MO, 63110; 3 Department of Microbiology, University of Ghana Medical School. College of Health Sciences, University of Ghana. PO Box GP 4236, Accra, Ghana; 4 Immunology Department. Noguchi Memorial Institute for Medical Research, College of Health Sciences, University of Ghana. Off Akilagpa Sawyerr Road. PO Box LG 581, Legon, Accra, Ghana; 5 Department of Epidemiology and Disease Control, School of Public Health, College of Health Sciences, University of Ghana, Legon, Accra, Ghana; 6 Department of Epidemiology, Noguchi Memorial Institute for Medical Research, College of Health Sciences, University of Ghana. Off Akilagpa Sawyerr Road. PO Box LG 581, Legon, Accra, Ghana

**Keywords:** COVID-19, data management, laboratory diagnosis, surveillance, pandemic

## Abstract

The COVID-19 pandemic caused by SARS-CoV-2 is an important subject for global health. Ghana experienced low-moderate transmission of the disease when the first case was detected in March 12, 2020 until the middle of July when the number of cases begun to drop. By August 24, 2020, the country's total number of confirmed cases stood at 43,622, with 263 deaths. By the same time, the Noguchi Memorial Institute for Medical Research (NMIMR) of the University of Ghana, the primary testing centre for COVID-19, had tested 285,501 with 28,878 confirmed cases. Due to database gaps, there were initial challenges with timely reporting and feedback to stakeholders during the peak surveillance period. The gaps resulted from mismatches between samples and their accompanying case investigation forms, samples without case investigation forms and vice versa, huge data entry requirements, and delayed test results. However, a revamp in data management procedures, and systems helped to improve the turnaround time for reporting results to all interested parties and partners. Additionally, inconsistencies such as multiple entries and discrepant patient-sample information were resolved by introducing a barcoding electronic capture system. Here, we describe the main challenges with COVID-19 data management and analysis in the laboratory and recommend measures for improvement.

**Funding:**

The work was supported by the Government of Ghana.

## Introduction

Effective surveillance systems with proper data collection and analysis during disease outbreaks play an integral role in supporting public health responses. The activities supported by an effective surveillance system might include event detection and characterization, enhanced surveillance, situational awareness, formal epidemiologic and laboratory investigations, identification and management of exposed persons, data collection and management and monitoring of the response itself and its effectiveness.^1^

Since data is the building block of effective response outcomes, the strategies regarding field investigation methods, laboratory detection, data collection, information to collect, and the tools for analysis are critical. Careful forecast of an event to predict the outbreak's trends and determine the degree of response depend on proper data management. Laboratory testing and reporting during disease outbreaks will also require a systematic appraisal of the methods and tools in use. Where needed, modify from one process to another for improvement. Laboratory data management practices also must remain adequate throughout the outbreak response.

Electronic health and surveillance systems have allowed remote staff to perform key duties such as data entry, cleaning, and analysis.^1^ Access by both laboratory, office and field staff to systematically collected data often simultaneously or in real-time improves support of the outbreak investigation.^2^, ^3^ Some of these innovations in data management have been deployed in Ghana during the current pandemic in response to the challenges brought on by the large number of samples that needed testing. This article traces the evolution of data management at the NMIMR during the initial COVID-19 outbreak response, the challenges faced, and lessons learned. We propose measures to improve data management in future pandemics.

### Overview of the initial COVID-19 data flow process

Suspected COVID-19 cases were identified through mandatory quarantine of travellers, passive case finding done at hospitals and clinics, Ghana Health Service (GHS) surveillance, contact tracers, and passive surveillance, which included walk-ins at health care facilities and testing centres. Case Investigation Forms (CIF) developed by the GHS were used to obtain clinical information and biodata of persons identified as suspects and contacts by the case/contact definition. This includes the name of patient, district, region, age, sex, date of onset, signs and symptoms, demographic information, name of health care facilities or hotel, date of sample collection, travel history and contact with an infected person. Nasopharyngeal and oropharyngeal swabs and sputum samples were collected into a transport medium and transported in cold boxes with their corresponding CIF to the laboratory at NMIMR.

Upon receipt, samples were matched with their corresponding CIF. A laboratory identification number (Lab ID) was assigned and written on the sample container and the CIF, along with the date received in the laboratory. The names, laboratory IDs, and the district and contact person at the district were recorded in laboratory notebooks. The information on the CIF which included name, Lab ID, suspected person's phone number, health district directorate, and phone number of the investigator) were entered into the database. The sample was then processed through RNA extraction and real-time polymerase chain reaction (RT-PCR). When the PCR results were ready, they were checked and verified by one of the supervisors (Research Fellows in the Virology Department), who, upon reviewing the PCR cycle thresholds, decided whether the test was positive or negative based on instructions that came with the test kits. The results were then recorded in a notebook beside their respective Lab IDs and sent to the data room, where they were recorded in the Excel sheet.

At this stage, results were ready to be transmitted to the surveillance officer whose phone number was on the CIF. Transmission of results was done by SMS text message from an official phone or by official email. In addition, daily collated results were sent to the GHS by email.

This system worked well until the Government of Ghana instituted the ‘enhanced contact tracing’ during the lockdown in the Greater Accra and Kumasi metropolis from March 30, 2020 to April 21, 2020. During this time, international travellers were not admitted into the country. The enhanced contact tracing meant mass testing of all in-coming travellers and all exposed persons in communities within these areas, which resulted in an increased number of confirmed COVID-19 cases. With the enhanced contact tracing, our centre received over 3000 samples per day from across the country. It soon became obvious that a mainly paper-based system will not suffice for multiple reasons. First, the paper-based system created a bottleneck in the sample processing laboratory since few people could enter Lab IDs into books at a time and make sure IDs were sequential. Second, after PCR results were entered into books containing Lab IDs, they had to be reconciled with the names in the Excel sheet, creating another bottleneck. Third, there was a backlog created for the entry of CIF forms into the database. The result of all these bottlenecks was that sometimes there were considerable delays in transmitting results and report summaries to health facilities and district health directorates in various regions, who needed the data for case management and contact tracing. To resolve this, we engaged over 30 secretarial staff at the NMIMR to enter the CIF information into the Excel database. Discussions and consultations ensued between the NMIMR, School of Public Health, Ghana Health Service and improvements were anticipated and implemented as detailed below.

### Transition to Microsoft Excel-based line list database

The initial paper-Excel hybrid was stopped. The Data Officers of the Department of Virology, NMIMR, used Excel sheets line list to enter the CIF, assign sample IDs and record PCR results. Upon receipt of the samples, CIF data entered in the laboratory were obtained by pen-drive and merged in the main database. A dedicated computer was set up where results were exported from the PCR runs on the Applied Biosystems (ABI) machines and emailed to the data team to be merged with the patient biodata. PCR results received in Excel sheets (with lab IDs but not names) were also smoothly assigned to their respective names in the data room.

Hence a system was established to transmit PCR results into the database seamlessly. Standard visualization techniques in Excel provided simple graphs summarizing data by time, place, and person. Initially, four data officers entered the data from different computers and information was harmonized at the end of the day. As more and more samples were collected, three teams were formed, rotating in a 12-hour shift to allow data to be entered round the clock. Therefore, the elimination of the paper system significantly improved work efficiency and transmission of results.

### Using REDCap to capture research-quality data

Since the data being captured in the Excel sheets were minimal, with the sole purpose of transmitting results to stakeholders, they were not useful for in-depth epidemiological data analysis and research. Therefore, we decided to re-capture the CIF information into the Research Electronic Data Capture (REDCap). REDCap is a web-based application used to capture data for clinical research and create databases and projects.^4^ It is a user-friendly secure platform compliant with the USA Health Insurance Portability and Accountability Act (HIPAA). Researchers can design their instruments for data capture into REDCap to generate a self-sufficient and secure database that can be used for normal data entry or for surveys across multiple distinct time points. REDCap is designed to provide a secure environment so that research teams can collect and store highly sensitive information. REDCap is now recommended as an ideal platform for data capture for different kinds of projects.^5^

Unlike the Excel line listing, which selected some variables from the case investigation form, all the information on the case investigation form, including all variables and results, are entered and suitable for detailed analysis for trends and hypothesis generation. Therefore, while the excel database only captured relevant information to share with stakeholders, the REDCap database captured additional in-depth and useful information for research.

### Transition to a barcode system: ArcGIS and SORMAs

Although the transition to electronic data capture helped tremendously, several problems remained. The most pressing was the amount of time spent by laboratory personnel to reconcile the CIF with samples. It became obvious that a barcode system where the CIF information was captured in the field was needed.

Once the CIF is captured in the field, the officers will bring samples with a line list. Upon scanning of the barcode on the samples, the suspected case will be identified in the system, given a lab ID, and processing will proceed smoothly. Initially, this system was piloted using a geopositioning information system (ArcGIS) software developed by colleagues in the Geography Department at the University of Ghana. ArcGIS could capture the location where the initial barcode was scanned, and the CIF information entered – the suspected case location. Such a system is perfect for contact tracing. In addition, by looking at the map, one could see where the hotspots of transmission were occurring. Eventually, this system was retired for the GHS Surveillance Outbreak Response Management Analysis System (SORMAS). Developed at the Helmholtz Centre for Infection Research in Germany, the system seeks to provide real-time electronic surveillance of outbreaks, especially in resource-limited settings^6^. This system works similarly to ArcGIS but is deployed nationwide. When the system is working optimally, district directors could log in to obtain their results. In addition, field officers can set their phones to receive alerts when results are entered into the system. With the SORMAS barcode system working, paper CIFs have gradually been phased out.

### Summary of Key Issues and Challenges in COVID-19 Data Management

The GHS Disease Surveillance Department used a well-structured standard case investigation form for data collection. However, the data collection was often incomplete on the CIF and, therefore, in the databases. Some of the records did not have information on the date of onset, sex, age, symptoms or nationality. This could be due to insufficient training of sample collectors, lack of proper supervision by surveillance officers or excessive workload. Many samples arrived in the laboratory without CIF, CIF without samples, and unmatched samples with CIF. In the early stages of the outbreak, samples were collected from travellers under mandatory quarantine and contacts of some travellers who did not go through the mandatory quarantine. Information at this time was better as compared to the peak of the outbreak.

At the beginning of the outbreak, a Microsoft Excel COVID-19 database was created to record information on each case. Due to the workload, substantially more staff were added to the teams to enter the data. Some of the data quality issues we encountered were basic data entry errors such as duplicated data, spelling mistakes, data in incorrect fields, and incomplete and erroneous data. Therefore, data supervision was intensified, and data cleaning and validation and quality checks were implemented at every stage to improve the quality and close or eliminate gaps.

As noted above, the deployment of SORMAS solved most of the field data quality issues. The samples were assigned barcodes, and the information on the case was scanned into the system. Once the sample reached the laboratory and was scanned, all the information on the sample appeared, and the test results could be added and made accessible to the system.

This online access system to the data and test results empowered the GHS field investigators with quick insight to make better and more informed decisions on the cases and contacts within their communities and jurisdictions. The GHS sent two disease surveillance officers to the NMIMR to help with data entry into SORMAS and address issues related to the system. The excellent collaboration between the GHS, NMIMR, and the University of Ghana's School of Public Health helped address the issues encountered in data management. Personnel from the GHS and SPH provided the needed training to the NMIMR to implement the electronic laboratory management systems described above.

Data cleaning was instituted to ensure that gaps were filled before results were reported. There were cases with inadequate information; the investigator on the CIF was contacted to provide the missing information to complete the data. Data cleaning identified cases without results, and the laboratory identification numbers were sorted to locate the samples and re-processed.

Due to the online channels and processes that were instituted, we overcame the key data management issues and challenges discussed above and improved upon the accuracy, validity, reliability and completeness of the data generated in the laboratory. We also worked closely and efficiently to manage and deploy daily reports by the laboratory to the Ministry of Health through the established platforms and a line list database. This dependence on the laboratory data never changed in this outbreak. It facilitated the timeliness and relevance of the data for decision-making until there were no lags in the laboratory data shared on the GHS COVID-19 platform.

## Recommendations

Based on the experiences and actions taken during the outbreak (as shown in Table 1), we recommend the following. First, proper and adequate training of field staff by the Ghana Health Service during disease outbreaks is key. Such field staff should be able to apply the tools to collect the appropriate data. Secondly, the trained surveillance officers from the GHS should closely supervise the sample and data collectors to ensure sound samples and information to the laboratories. Thirdly, laboratory staff training and appropriate tools needed for data management should be made available to the laboratories by the Ministry of Health. Finally, the Ministry and its international partners should equip laboratories with data dissemination tools to ensure timely and accurate reporting of test results to key stakeholders.

**Table 1:** Key challenges in laboratory data management and action taken to overcome them

Key challenges	Actions taken
Incomplete data collection on the CIF	A continuous reminder of the Head of Disease Surveillance to advise the samples collectors helped improve the situation.
Some of the records did not have vital information on date of onset, sex, age, symptoms or nationality	District Directors were called to provide the missing information to complete the CIF.
Many samples arrived in the laboratory without CIF, CIF without samples as well as unmatched samples with CIF	Efforts were made to sort all samples and assigned them to their respective CIF.
Inadequate information on samples collected from travellers under mandatory quarantine and their contacts at the peak of the outbreak.	Investigators were made to go back to locate and collect the relevant information needed.
Basic data entry errors such as duplicated data, spelling mistakes, data in incorrect fields, incomplete and erroneous data were some of the data quality issues encountered	Data supervision was intensified. Data cleaning, validation, and quality checks were implemented at every stage (receipt, entry, analysis, reporting) to improve the quality and close or eliminate gaps.
There were delays in sending out reports from the laboratory during the peak period.	Movement from paper-based records to electronic and entries and SORMAS solved this issue.
The country had no stock of reagents as at the onset of the outbreak.	Reagents were solicited from projects and other departments to kick start the sample processing. There should be a national stockpile of reagents and consumables to respond to disease outbreaks.

## Conclusion

Proper laboratory data management for SARS-CoV-2 disease is important as the results from the laboratory are used by the GHS for decision making. During the early stage of the COVID-19 outbreak, field and laboratory data management was poor, but these improved in the course of the outbreak due to the data measures implemented. Several challenges, including sample labelling, filling of CIF and data handling, were encountered. Still, with improved data management tools and systems, timely and accurate results were reported daily to the GHS for action.

## Data Team

Michael A.P. Ntiri, Anthony Twumasi Boateng, Richard Akuffo, Stephen Nyarko, Richard Obeng and Kojo Mensah Sedzro
